# Aberrant Resting-State Functional Connectivity Associated With Callous-Unemotional Traits From Late Childhood Through Late Adolescence

**DOI:** 10.1016/j.bpsgos.2026.100756

**Published:** 2026-05-12

**Authors:** Emily C. Kemp, Herry Patel, Sofia Estradaflores, Zeying Du, Isabel R. Aks, Fiona A. Ralston, William E. Pelham

**Affiliations:** a Department of Psychiatry, University of California San Diego, San Diego, California; b School of Public Health, Yale University, New Haven, Connecticut; c Department of Psychology, University of Michigan, Ann Arbor, Michigan

**Keywords:** Callous-unemotional (CU) traits, Default mode network (DMN), Frontoparietal network (FPN), Resting-state functional connectivity, Salience network (SN), Tri-network model

## Abstract

**Background:**

Callous-unemotional (CU) traits are a marker of deficient socioemotional processing linked to severe conduct problems. Although resting-state studies implicate altered large-scale network organization in CU traits, few have comprehensively examined connectivity within and between the default mode network (DMN), salience network (SN), and frontoparietal network (FPN), and none have done so across development in large samples.

**Methods:**

Using data from the Adolescent Brain Cognitive Development Study (*N* = 11,868; 48% female), we examined associations between CU traits and resting-state functional connectivity (rsFC) within and between the DMN, FPN, and SN with repeated time points (4) across youths spanning ages 9 to 18 years. Linear mixed-effects models were used to assess the main effects of CU traits along with moderators of age and sex, and sensitivity analyses included covariates for co-occurring psychopathology.

**Results:**

CU traits were associated with reduced within-DMN rsFC. No interactions between CU traits and sex were found, but an interaction between CU traits and age emerged, showing a negative association between CU traits and DMN-SN rsFC that was evident in mid-to-late adolescence, with higher-CU youths showing an age-related decline in DMN-SN rsFC.

**Conclusions:**

These findings suggest that youth with elevated CU traits exhibit atypical development of resting-state connectivity in networks crucial for empathy and salience processing. Specifically, youth with elevated CU traits showed persistent DMN hypoconnectivity and a later-emerging but accelerated age-related segregation of DMN-SN connectivity during adolescence. This pattern may reflect delayed yet ultimately exaggerated differentiation of socioaffective networks, highlighting adolescence as a developmental window for interventions targeting socioemotional functioning.

## Plain Language Summary

Youth with elevated callous-unemotional traits lack empathy and remorse, placing them at risk for severe, persistent antisocial behavior. However, much remains unknown about how their brains develop through childhood and adolescence. We studied brain scans from nearly 12,000 youths ages 9–18 years collected multiple times over several years. Youths with higher callous-unemotional traits showed weaker connections in a brain network responsible for understanding others’ feelings and reflecting on one’s emotions. These differences appeared as early as age 9 and persisted through 18, with additional between-network changes emerging specifically during adolescence. Identifying when and where brain differences occur could help target prevention efforts.

Callous-unemotional (CU) traits, defined by deficient remorse, empathy, and affect (1), mark a distinct neuroaffective profile among youth with conduct problems (2,3) and are associated with numerous problematic outcomes (e.g., harm to others, legal involvement) (4,5). Further, CU traits that have been shown to onset in early childhood (6) increasingly stabilize from middle childhood through adolescence (1,7) and increase risk for antisocial personality disorder or psychopathy in adulthood (8, 9, 10). These developmental patterns suggest that childhood and adolescence are critical periods for the development of CU-related brain alterations (1,11). Sex differences in prevalence and heritability have also been noted, and some studies report sex-specific neural correlates (12), but evidence is inconsistent (13, 14, 15) and research in females remains limited.

Menon’s tri-network model of psychopathology (16) provides a useful framework for investigating whether CU traits are related to reductions in intrinsic network connectivity. This model focuses on 3 large-scale brain networks proposed to work in concert with one another and correlate with all psychiatric disorders. These networks include 1) the default mode network (DMN), implicated in self-referential thought and sociocognitive processes such as empathy, moral reasoning, and autobiographical memory; 2) the frontoparietal network (FPN), involved in higher-level cognitive control, goal-directed decision making, and problem solving; and 3) the salience network (SN), needed for detecting and integrating salient stimuli and in switching between the DMN and FPN in response to environmental change or demands.

From childhood through adolescence, these networks undergo significant maturation. Within-network resting-state functional connectivity (rsFC) typically increases while between-network connectivity decreases as networks become more segregated and specialized (12). This segregation is thought to reflect increasingly efficient coordination between internally (DMN) and externally (FPN/SN) oriented systems as cognitive and socioemotional regulation mature in response to increasingly complex environmental demands. Importantly, these changes are not uniform across individuals. For instance, females generally show more mature, enhanced connectivity, particularly within the DMN, and earlier in development (13). Together, these findings indicate that adolescence is a critical window of neural specialization, during which emerging individual and sex differences may become more visible as networks differentiate.

Growing evidence suggests that CU traits are associated with alterations in rsFC as defined by Menon’s tri-network model. For example, a recent cross-sectional study of over 9500 preadolescents from the Adolescent Brain Cognitive Development (ABCD) Study found that higher CU traits were associated with reduced connectivity within the DMN (14). This within-DMN hypoconnectivity could indicate diminished self-referential processing, introspection, and mentalizing abilities, potentially explaining deficient remorse and empathy (i.e., youth may be less able to link observable consequences to their actions or integrate the observed experiences of others to the self). Hypoconnectivity within the FPN has also been noted in cross-sectional studies of youths with poor cognitive control (15), and one resting-state functional magnetic resonance imaging (rs-fMRI) study of 84 adolescents reported that higher CU traits were associated with lower FPN density (17). While the link between CU traits and the FPN is less clear, reduced FPN integrity may help explain the higher levels of impulsivity and disinhibition often observed in youth with elevated CU traits (18,19). Furthermore, weaker between-network rsFC between the FPN and DMN has been linked to poorer top-down control and greater reactive aggression in 2 cross-sectional studies of adolescents with CU traits (17,20). Regarding the SN, Caldwell *et al.* (21) found that, in a sample of nearly 300 incarcerated adolescent males assessed at a single time point, CU traits were associated with reduced connectivity within the SN. This aligns with other evidence that core SN regions (e.g., anterior insula) are associated with reduced functioning in youth with elevated CU traits (22). Similarly, a study by Dugré and Potvin (23) that drew data from nearly 1500 adolescents from the Healthy Brain Network reported that CU traits were associated with altered connectivity between the amygdala, a key node of the limbic system implicated in the broader SN, and other brain regions (e.g., thalamus), depending on the level of co-occurring anxiety. Further, emerging developmental network models suggest that antisocial youth without elevated CU traits may show relatively greater executive network disruption, whereas high-CU phenotypes may be more selectively associated with alterations in socioaffective circuitry (7,24). Together, these findings underscore the potential of rsFC to reveal a clinically relevant neural signature of CU traits.

Despite advances in the field, several gaps remain. Namely, most rsFC studies of CU traits have been limited by 1) cross-sectional designs with single time point assessments that narrowly tap age, largely during mid-to-late adolescence, that cannot capture developmental variation (25); 2) variable sample sizes at risk of yielding unreliable results (26); 3) samples drawn from justice-involved or clinically severe populations, which provide critical insight into later-stage disorder expression but may reflect more extreme CU phenotypes compared with community-based samples; and 4) a failure to simultaneously examine all 3 networks of the tri-network model, providing only partial evidence of intrinsic neural functioning underlying CU traits. While a recent study by Umbach and Tottenham (14) addressed some of these issues (i.e., sample size), they assessed participants only at baseline, providing a view of a relatively narrow developmental window (ages 9–11 years), and explicitly focused on the DMN.

The current study addresses these gaps by leveraging the large, diverse, and longitudinal ABCD dataset to examine associations between CU traits and rsFC within and between the DMN, SN, and FPN from late childhood (age 9) through late adolescence (age 18). This wide developmental window allowed us to test whether CU-rsFC associations are stable across youths or emerge during adolescence, a period of rapid network specialization. Examining multiple networks simultaneously also enabled us to identify whether CU traits reflect broad disruptions in large-scale network organization or more selective alterations. Finally, we evaluated moderation by age and sex, given known developmental and epidemiological differences. Based on prior work, we hypothesized that CU traits would be associated with reduced within-DMN connectivity. We further explored whether between-network connectivity varied as a function of CU traits and whether these associations differed by age or sex.

## Methods and Materials

### Sample and Design

The current study used brain and behavioral data from the ABCD Study. Youths were recruited at ages 9 and 10 years (mean = 9.96 years, 48% female) between 2016 and 2018 from 21 study sites across the United States, resulting in a total baseline sample of 11,880 youths. Recruitment was school based and epidemiologically guided [see Garavan *et al.* (27) for study details]. Exclusion criteria were minimal and primarily focused on excluding participants who were unable to comfortably and accurately complete assessments (e.g., safety contraindications for MRI) (27). After the baseline visit, the youths and their participating parents were followed with annual assessments and rs-fMRI data were collected every 2 years. Data for the current study were drawn from the ABCD 6.1 Release (see https://abcdstudy.org/scientists/data-sharing/ and https://www.nbdc-datahub.org/), which includes data from the baseline visit through the 6-year follow-up. See Table 1 for more detailed sample descriptives (e.g., youth race and ethnicity, household income, parent education, and marital status).

**Table 1:** Sample Characteristics and Descriptives for Variables of Interest

Variable	Baseline, *N* = 11,868	2-Year Follow-Up, *n* = 10,973	4-Year Follow-Up, *n* = 9739	6-Year Follow-Up, *n* = 5056
Age, Years	9.96 (0.62)	12.07 (0.67)	14.17 (0.71)	16.07 (0.66)
Female, %	48%	–	–	–
Parent Education Level, Years	16.59 (2.77)	–	–	–
Parent Marital Status, Median Selected Option	Married	–	–	–
Household Annual Income, Median Selected Option	$75,000 to $100,000	–	–	–
Race/Ethnicity				
Asian	2%	–	–	–
Black	15%	–	–	–
Hispanic	21%	–	–	–
Other	10%	–	–	–
White	52%	–	–	–
CU Traits, Sum Score	0.90 (1.39)	0.98 (1.45)	1.14 (1.56)	1.10 (1.54)
Within-Network Connectivity				
DMN	0.24 (0.06)	0.25 (0.06)	0.26 (0.06)	0.26 (0.06)
FPN	0.21 (0.06)	0.22 (0.06)	0.23 (0.06)	0.24 (0.06)
SN	0.39 (0.13)	0.38 (0.12)	0.37 (0.12)	0.38 (0.12)
Between-Network Connectivity				
DMN-FPN	0.05 (0.04)	0.05 (0.04)	0.05 (0.05)	0.06 (0.05)
DMN-SN	0.07 (0.06)	0.07 (0.06)	0.08 (0.06)	0.08 (0.07)
FPN-SN	0.08 (0.07)	0.09 (0.06)	0.10 (0.07)	0.10 (0.07)
Pubertal Stage, Median Tanner Stage	Early	Mid	Late	Late
Anxiety Symptoms	2.06 (2.43)	1.81 (2.32)	1.70 (2.31)	1.51 (2.20)
ADHD Symptoms	2.63 (2.97)	2.29 (2.77)	2.04 (2.63)	1.76 (2.43)
CD Symptoms	1.28 (2.36)	1.14 (2.25)	1.10 (2.26)	1.02 (2.21)

Values are presented as mean (SD) or %, unless otherwise specified.

ADHD, attention-deficit/hyperactivity disorder; CD, conduct disorder; CU, callous-unemotional traits; DMN, default mode network; FPN, frontoparietal network; SN, salience network.

### Measures

#### CU Traits

CU traits were assessed using a validated parent-report scale (28) that has been used in at least one dozen prior studies of CU traits in the ABCD sample, including studies of structural and functional brain reactivity differences related to CU traits (29,30). This scale includes one item, “doesn’t seem to feel guilty after misbehaving,” from the Child Behavior Checklist (CBCL) (31) and 3 reversal items from the Strengths and Difficulties Questionnaire (SDQ) [i.e., “considerate of others’ feelings,” “helpful if someone is hurt or upset,” and “offers to help others” (32)]. All items were scored using a 3-point scale (0 = not true; 1 = somewhat/sometimes true; 2 = very, often, or certainly true). After reverse scoring the SDQ items, the 4 items were summed to create a single total score, with mean proration applied to handle missing data for any participants with at least 3 item responses available. Higher scores reflect higher CU traits. Internal consistency was acceptable across all time points (αs = 0.75–0.79). Additional validity analyses are reported in the Supplement.

#### Resting-State FC

Gordon network correlations (33) were used to assess within- and between-network connectivity among the DMN, SN, and FPN, based on Menon’s tri-network model (16), at baseline and 2, 4, and 6-year time points.

rsFC was assessed as part of a larger neuroimaging battery for a total of approximately 20 minutes (34, 35). During each scan, participants were instructed to lie still with their eyes open while staring at a fixation cross (+) presented on a screen within the scanner. Individual scans were excluded based on quality control checks performed by the Data Analysis, Informatics & Resource Center, which consisted of excessive head motion or incomplete runs (for more information, see https://abcdstudy.org/study-sites/daic/). Preprocessing was conducted using a state-of-the-art pipeline described by Hagler *et al.* (35), which includes artifact correction, brain segmentation, and surface-based functional connectivity processing.

rsFC data were analyzed using a seed-based correlation approach tailored for cortical surface analysis (35). Average time series were extracted for anatomically and functionally defined regions of interest (ROIs). Gordon parcellation ROIs were organized into different networks, including the DMN, FPN, and SN (33). Correlation values between ROI pairs were calculated and then Fisher transformed into *z* statistics. They were then averaged across within- and between networks to assess the strength of the network correlations.

#### Covariates

A set of standard covariates, including youth age (continuous), sex at birth, pubertal status, MRI scanner type, and head motion, were included in all analyses. Pubertal status was assessed at each study time point with approximated Tanner stages (e.g., early, mid, late) evaluated with the Pubertal Development Scale (36) and menstrual cycle histories reported by the youth and parent [see Cheng *et al.* (37) for more details].

In addition, sensitivity analyses included a set of covariates reflective of commonly co-occurring psychiatric symptoms to adjust for potential confounding. The question of what exactly to adjust for is not an easy one to answer, but these tests were included for estimate comparison and robustness. Each of these covariates were assessed using parent-reported total scores from the CBCL at each study time point. In the first step, we included attention-deficit/hyperactivity disorder (ADHD) symptoms using scores from the CBCL DSM-5 ADHD problems subscale and anxiety symptoms, the levels of which are shown to characterize distinct CU variants, with scores from the DSM-5 anxiety problems subscale (31). Finally, to further parse the unique associations between CU traits and rsFC, we covaried for co-occurring but non-overlapping conduct disorder (CD) symptoms, which sometimes present suppressor effects (38,39) and may be more easily observed by parents, using scores from the CBCL DSM-5 CD symptoms subscale (31).^a^

### Data Analytic Plan

Data were analyzed in R version 4.5.2 using the lme4 package. We modeled the effects of CU traits on within- and between-rsFC of 3 networks in Menon’s tri-network model across 4 biennial assessments, spanning ages 9 to 18 years. For all analyses, mixed-effects models were fitted using maximum likelihood estimation. All models included a hierarchical random effects structure to account for data dependencies: Participants were nested within families, which were nested within ABCD Study sites. Family ID was assigned to all members of the same household, including siblings and twins, such that this nesting structure appropriately modeled shared genetic and environmental variance without requiring sibling exclusion or random selection. This approach accounts for both within-family clustering and between-site variability in recruitment and scanning protocols.

Fixed effects consisted of the assessment time point, CU traits, and standard covariates: youth age, biological sex, pubertal status, MRI scanner, and head motion. Age was treated as a time-varying covariate to account for developmental differences at each scan, thereby allowing us to test whether CU associations with rsFC varied by age without imposing individual growth-curve trajectories, which were not the focus of our hypotheses.

To test whether associations between CU traits and rsFC varied by age or sex, we added interaction terms in separate sets of models. Specifically, CU traits × age interactions tested whether CU-rsFC associations strengthened or weakened across development, while CU traits × sex interactions examined whether effects differed between males and females. Age and sex were retained as main effect covariates in all models, regardless of whether their interactions with CU traits were tested.

Sensitivity analyses assessed the robustness and specificity of CU associations with rsFC by including additional covariates for commonly co-occurring psychiatric symptoms. These models adjusted for ADHD, anxiety, and CD symptoms from the CBCL. Supplemental sensitivity models separately adjusted for 1) aggression problems from the CBCL, 2) lifetime childhood-onset CD diagnosis from the Kiddie Schedule for Affective Disorders and Schizophrenia, and 3) total cortical surface area. Results from all supplemental sensitivity models are reported in Tables S1 and S2.

Benjamini-Hochberg false discovery rate (FDR) correction (40) was applied to primary hypothesis tests (Table 2) across the family of 18 tests (6 rsFC outcomes × 3 inferential tests: main CU effect, CU × age, CU × sex). Only effects surviving FDR correction (*q* values < .05) are reported as significant in primary analyses, and we report both uncorrected *p* values and FDR-corrected *q* values (41). Sensitivity analyses with additional psychiatric covariates (i.e., ADHD, anxiety, CD symptoms) (Tables 3 and 4) were treated as conditional robustness checks evaluating whether primary effects remain under more stringent covariate control and were not subject to FDR correction (i.e., unadjusted *p* values are reported).

**Table 2:** Main Effects of CU Traits and Their Interactions With Either Youth Age or Sex on rsFC Outcomes

Outcome	Predictor	*B* (SE)	β (SE)	*p* Value	*q* Value	*n*_Youth_	*n*_Observations_
DMN	CU traits	−0.0009 (0.0003)	−0.0224 (0.0063)	<.001	.002∗	10,530	22,666
	CU × age	−0.0002 (0.0000)	−0.0035 (0.0023)	.127	.212		
	CU × female	−0.0000 (0.0005)	−0.0003 (0.0127)	.979	.979		
FPN	CU traits	0.0003 (0.0003)	0.0070 (0.0064)	.278	.767	10,530	22,666
	CU × age	−0.0000 (0.0000)	−0.0007 (0.0023)	.767	.767		
	CU × female	0.0002 (0.0005)	0.0046 (0.0130)	.721	.767		
SN	CU traits	0.0005 (0.0006)	0.0062 (0.0068)	.362	.812	10,530	22,666
	CU × age	−0.0000 (0.0002)	−0.0006 (0.0025)	.812	.812		
	CU × female	0.0006 (0.0011)	0.0067 (0.0137)	.627	.812		
DMN-FPN	CU traits	0.0002 (0.0002)	0.0068 (0.0068)	.313	.878	10,530	22,666
	CU × age	−0.0000 (0.0000)	−0.0007 (0.0025)	.793	.878		
	CU × female	0.0000 (0.0004)	0.0021 (0.0137)	.878	.878		
DMN-SN	CU traits	−0.0003 (0.0003)	−0.0066 (0.0067)	.329	.549	10,530	22,666
	CU × age	−0.0003 (0.0001)	−0.0067 (0.0025)	.007	.036∗		
	CU × female	−0.0002 (0.0006)	−0.0039 (0.0136)	.774	.774		
FPN-SN	CU traits	−0.0002 (0.0003)	−0.0039 (0.0067)	.554	.557	10,530	22,666
	CU × age	−0.0000 (0.0001)	−0.0018 (0.0024)	.467	.557		
	CU × female	0.0007 (0.0006)	0.0152 (0.0134)	.257	.557		

Unstandardized (*B*) and standardized (β) estimates and SE are presented for either the main effect of CU traits or the interaction between CU traits and age or CU traits and sex (female) modeled separately. All steps included standard covariates of youth age, sex, pubertal status, study time point, magnetic resonance imaging scanner type, and head motion. Random effects included participant ID nested within family ID nested within assessment site.

∗Values that survived false discovery rate correction at *q* < .05.

CU, callous-unemotional traits; DMN, default mode network; FPN, frontoparietal network; rsFC, resting-state functional connectivity; SN, salience network.

**Table 3:** Main Effects of CU Traits on rsFC Outcomes in the Presence of Comorbidity Covariates

Outcome	Covariates	*B* (SE)	β (SE)	*p* Value	*n*_Youth_	*n*_Observations_
DMN	Standard + ADHD, anxiety	−0.0008 (0.0003)	−0.0182 (0.0064)	.005∗	10,530	22,660
FPN		0.0005 (0.0003)	0.0115 (0.0066)	.083		
SN		0.0006 (0.0006)	0.0069 (0.0070)	.325		
DMN-FPN		0.0002 (0.0002)	0.0064 (0.0070)	.361		
DMN-SN		−0.0004 (0.0003)	−0.0080 (0.0070)	.253		
FPN-SN		−0.0000 (0.0003)	−0.0009 (0.0069)	.900		
DMN	Standard + ADHD, anxiety, CD symptoms	−0.0008 (0.0003)	−0.0190 (0.0069)	.006∗	10,530	22,660
FPN		0.0004 (0.0003)	0.0086 (0.0070)	.216		
SN		0.0001 (0.0006)	0.0015 (0.0073)	.838		
DMN-FPN		0.0003 (0.0002)	0.0083 (0.0076)	.270		
DMN-SN		−0.0003 (0.0003)	−0.0078 (0.0074)	.296		
FPN-SN		−0.0002 (0.0003)	−0.0051 (0.0073)	.485		

Unstandardized (*B*) and standardized (β) estimates and SE are presented for the main effect of CU traits. Standard covariates included youth age, sex, pubertal status, study time point, magnetic resonance imaging scanner type, and head motion. Random effects included participant ID nested within family ID nested within assessment site.

∗Significant at *p* < .05.

ADHD, attention-deficit/hyperactivity disorder; CD, conduct disorder; CU, callous-unemotional traits; DMN, default mode network; FPN, frontoparietal network; rsFC, resting-state functional connectivity; SN, salience network.

**Table 4:** Interaction Effects of CU Traits and Either Youth Age or Sex on rsFC Outcomes in the Presence of Comorbidity Covariates

Predictor	Outcome	Covariates	*B* (SE)	β (SE)	*p* Value	*n*_Youth_	*n*_Observations_
CU × Age	DMN	Standard + ADHD, anxiety	−0.0002 (0.000)	−0.0038 (0.0023)	.100	10,530	22,660
CU × Female			−0.0000 (0.0005)	−0.0013 (0.0127)	.919		
CU × Age	FPN		−0.0000 (0.000)	−0.0011 (0.0023)	.627		
CU × Female			0.0001 (0.0005)	0.0036 (0.0130)	.782		
CU × Age	SN		−0.0000 (0.0002)	−0.0006 (0.0025)	.797		
CU × Female			0.0006 (0.0011)	0.0074 (0.0137)	.591		
CU × Age	DMN-FPN		−0.0000 (0.000)	−0.0006 (0.0025)	.804		
CU × Female			0.0000 (0.0004)	0.0023 (0.0137)	.866		
CU × Age	DMN-SN		−0.0003 (0.0001)	−0.0066 (0.0025)	.007∗		
CU × Female			−0.0002 (0.0006)	−0.0034 (0.0136)	.805		
CU × Age	FPN-SN		−0.0000 (0.0001)	−0.0021 (0.0024)	.392		
CU × Female			0.0007 (0.0006)	0.0152 (0.0134)	.257		
CU × Age	DMN	Standard + ADHD, anxiety, CD symptoms	−0.0002 (0.0001)	−0.0037 (0.0023)	.101	10,530	22,660
CU × Female			−0.0000 (0.0005)	−0.0010 (0.0124)	.936		
CU × Age	FPN		−0.0000 (0.0001)	−0.0011 (0.0023)	.642		
CU × Female			0.0002 (0.0005)	0.0041 (0.0127)	.744		
CU × Age	SN		−0.0000 (0.0002)	−0.0005 (0.0024)	.826		
CU × Female			0.0007 (0.0011)	0.0084 (0.0133)	.529		
CU × Age	DMN-FPN		−0.0000 (0.0001)	−0.0006 (0.0025)	.793		
CU × Female			0.0001 (0.0004)	0.0019 (0.0137)	.891		
CU × Age	DMN-SN		−0.0003 (0.0001)	−0.0066 (0.0025)	.007∗		
CU × Female			−0.0001 (0.0006)	−0.0034 (0.0135)	.803		
CU × Age	FPN-SN		−0.0001 (0.0001)	−0.0020 (0.0024)	.409		
CU × Female			0.0007 (0.0006)	0.0161 (0.0133)	.226		

Unstandardized (*B*) and standardized (β) estimates and SE presented for interactions between CU traits and age or sex (female). Standard covariates included youth age, sex, pubertal status, study time point, magnetic resonance imaging scanner type, and head motion. Random effects included participant ID nested within family ID nested within assessment site.

∗Significant at *p* < .05.

ADHD, attention-deficit/hyperactivity disorder; CD, conduct disorder; CU, callous-unemotional traits; DMN, default mode network; FPN, frontoparietal network; rsFC, resting-state functional connectivity; SN, salience network.

## Results

**Figure 1:**
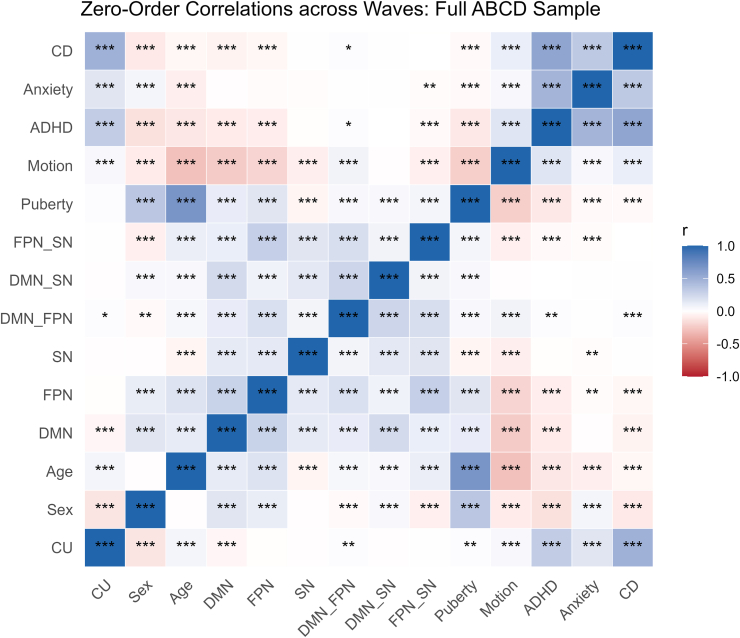
Zero-order correlations across waves in the Adolescent Brain Cognitive Development (ABCD) Study sample. Puberty represents approximated Tanner stage (higher = later in pubertal development); motion represents average head motion during resting-state task only. ∗*p* < .05, ∗∗*p* < .01, ∗∗∗*p* < .001. ADHD, attention-deficit/hyperactivity disorder; CD, conduct disorder; CU, callous-unemotional traits; DMN, default mode network; FPN, frontoparietal network; SN, salience network.

Figure 1 shows the bivariate (zero-order) correlations between variables of interest, including the predictor CU traits; standard covariates of age, sex, pubertal status, and head motion; primary psychiatric symptom covariates of ADHD, anxiety, and CD symptoms; and rsFC outcomes.

### Main Analytic Model Results

#### Within-Network rsFC

Table 2 shows tests of rsFC within each network. CU traits were associated with decreased DMN rsFC (*B* = −0.0009, β = −0.0224, *p* < .001, *q* = .002). CU traits were not associated with FPN or SN rsFC, and no interactions between CU traits and either age or sex were observed for any rsFC outcome.

#### Between-Network rsFC

Table 2 shows tests of rsFC between each network. CU traits were not associated with any between-network rsFC (*p*s > .05) on their own, and no interaction effects for CU traits and sex were found. However, there was a significant interaction between CU traits and age associated with decreased DMN-SN rsFC (*B* = −0.0003, β = −0.0067, *p* = .007, *q* = .036).^b^

**Figure 2:**
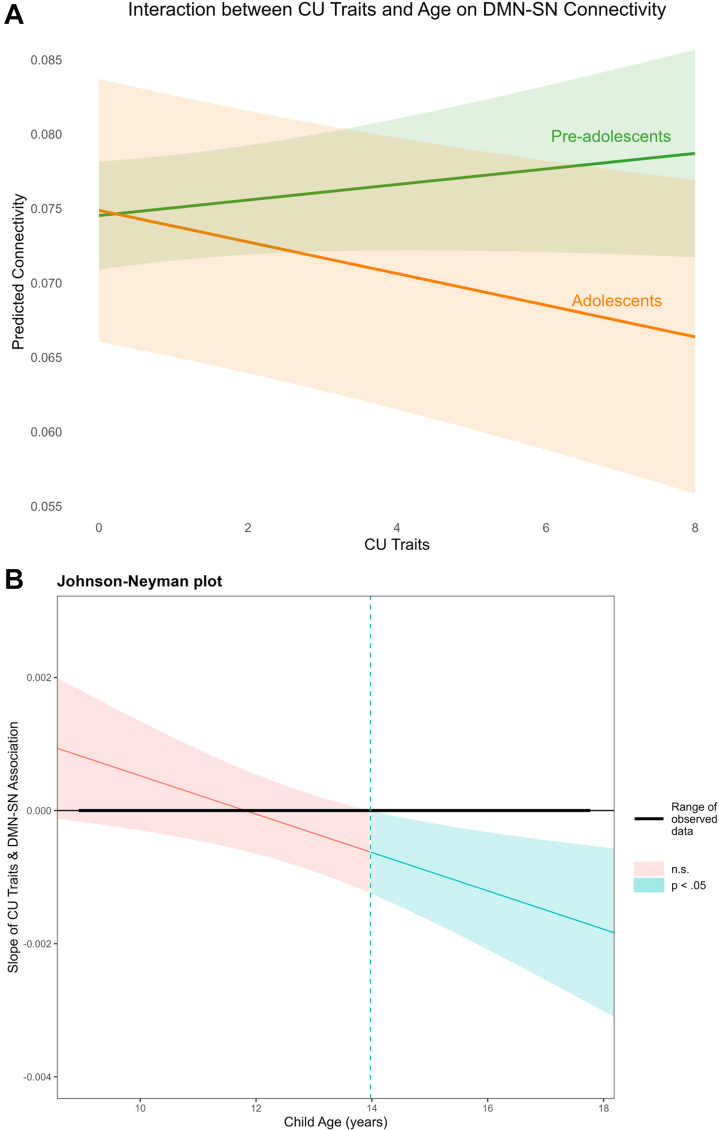
**(A)** Interaction between callous-unemotional (CU) traits and age on resting-state functional connectivity (rsFC) between the default mode network (DMN) and salience network (SN). Preadolescents = 8 to 12 years and adolescents = 13 to 18 years. **(B)** Johnson-Neyman plot. Regions of significance (shaded blue outside the dotted vertical lines) for the interaction effect of CU traits and age on rsFC between the DMN and SN indicate that the negative association between CU traits and rsFC becomes statistically significant at age ≥14 years. The range of observed ages is 8.96 to 17.75 years. n.s., nonsignificant.

**Figure 3:**
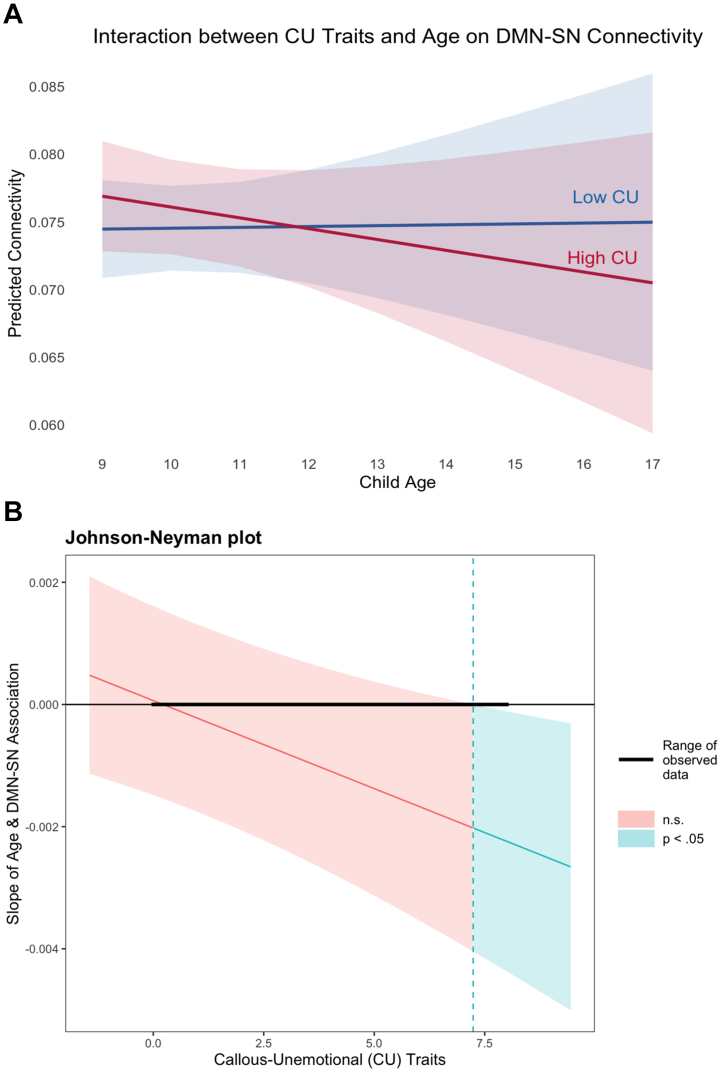
**(A)** Interaction between CU traits and age on resting-state functional connectivity (rsFC) between the default mode network (DMN) and salience network (SN). Low CU indicates CU total score at or below the 10th percentile (i.e., 0); high CU indicates CU total score at or above the 90th percentile (i.e., 3). **(B)** Johnson-Neyman plot. Regions of significance (shaded blue) for the interaction between CU traits and age in their associations with rsFC between the DMN and SN indicate that the negative association between age and rsFC becomes statistically significant at a CU score of ≥7.24 (i.e., <1% of the total sample or sample at any time point). The range of observed CU total scores = 0 to 8. n.s., nonsignificant.

Figure 2A plots this interaction between CU traits and age on DMN-SN rsFC and shows a negative association between CU traits and DMN-SN rsFC present during adolescence (i.e., simple slope: *B* = −0.0009, SE = 0.0004, 95% CI [−0.0017 to −0.0002]) but not preadolescence (i.e., simple slope: *B* = 0.0004, SE = 0.0004, 95% CI [−0.0004 to 0.0011]). In addition, Figure 2B shows that the association between CU traits and lower DMN-SN rsFC becomes significant at age ≥14. Figure 3A depicts the same interaction but with CU traits as the moderator and shows DMN-SN rsFC starts higher in youths with higher CU traits but then declines during adolescence (*B* = −0.0008, SE = 0.0008, 95% CI [−0.0024 to 0.0008]), as opposed to youths with low or no CU traits who appear to show stable patterns of connectivity (*B* = 0.0000, SE = 0.0008, 95% CI [−0.0015 to 0.0016]). Figure 3B shows that this negative association between age and DMN-SN rsFC becomes significant at a CU score of ≥7.24. This score represents an extreme end of the CU spectrum (range = 0–8) observed in this sample.

### Sensitivity Analyses

Table 3 shows the main effects of CU traits on rsFC outcomes, with ADHD and anxiety included as covariates. The main effect of CU traits on decreased within-DMN rsFC remained significant (*B* = −0.0008, β = −0.0182, *p* = .005), indicating this association was not accounted for by overlapping symptom dimensions. The inclusion of CD symptoms as an additional covariate did not alter this result (*B* = −0.0008, β = −0.0190, *p* = .006).^c^ Like before, no other within- or between-rsFC associations with CU traits reached significance.

Table 4 shows interaction effects between CU traits and either age or sex in the presence of comorbidity covariates. The interaction between CU traits and age on DMN-SN rsFC persisted (*B* = −0.0003, β = −0.0066, *p* = .007) after adjusting for ADHD and anxiety. This pattern was unchanged when CD symptoms were added (*B* = −0.0003, β = −0.0066, *p* = .007), suggesting that the developmental moderation of CU traits on DMN-SN rsFC is robust. Like before, all other CU interactions remained nonsignificant.^d^

## Discussion

In a nationwide community sample (*N* = 11,868) spanning ages 9 to 18 years, we found that youths with elevated CU traits, regardless of sex, exhibited atypical patterns of rsFC in networks crucial for empathy, salience processing, and healthy socioemotional functioning. These findings fill key gaps, clarify prior cross-sectional work, and provide novel results that could only be detected with data spanning a wide developmental range. First, by leveraging the largest sample with repeated rsFC assessments to date, and by doing so in an epidemiologically informed cohort in which CU traits vary dimensionally rather than restricted to clinical extremes, we were able to show that CU-related neural alterations are not static. Rather, they appear to include early-emerging, developmentally stable DMN hypoconnectivity and later-emerging, adolescence-specific acceleration in DMN-SN segregation. Second, by simultaneously examining within- and between-network connectivity across all 3 hubs of the tri-network model, we provide a more complete picture of how CU traits map onto large-scale neural systems, demonstrating selective disruption in socioaffective networks (DMN and DMN-SN). Finally, by explicitly modeling interactions between CU traits and both age and sex, we were better able to delineate when CU-related neural differences are observed and to demonstrate their similarity across sex. Together, these contributions offer a developmentally grounded, neurobiologically informed model of underlying deficits in CU traits and underscore adolescence as a key window in which atypical socioaffective network development may consolidate, highlighting critical opportunities for intervention.

### DMN Hypoconnectivity

The association between CU traits and within-DMN hypoconnectivity replicates and extends prior evidence (14) that CU traits are related to reduced intrinsic coherence within networks underlying self-referential and sociocognitive processing. Importantly, within-DMN rsFC hypoconnectivity was present across a wide developmental span (i.e., ages 9–18), with no observed moderation by age or sex, and was found to be robust against the inclusion of highly co-occurring symptom dimensions and supplemental sensitivity tests. Thus, reduced DMN functional connectivity appears to be a developmentally persistent, non–sex-specific neural correlate of CU traits and may present a clinically meaningful biomarker.

### Between-Network Segregation

Building on this, our most novel finding is the developmental moderation of DMN-SN connectivity. While no association between CU traits and DMN-SN connectivity was observed in preadolescence (ages 9–12), a robust negative association emerged and strengthened by mid-to-late adolescence (age ∼14), indicating greater network segregation with increasing age. This pattern may best be interpreted as reflecting an initial developmental delay in network differentiation (i.e., higher-CU youths starting at higher [less segregated] levels of DMN-SN connectivity than low-CU youths during preadolescent years), followed by an accelerated decoupling during adolescence. That is, early in development, youths with elevated CU traits may show less differentiation between the DMN and SN, signaling immature coordination between internal (DMN) and salience-driven (SN) processes, before undergoing an exaggerated phase of segregation later. This trajectory may represent an atypical pacing, rather than direction, of normative network maturation. This interpretation is broadly consistent with emerging large-scale structural neuroimaging evidence suggesting heterogeneity in neurodevelopmental timing across antisocial subtypes. That is, a recent mega-analysis from the Enhancing Neuro Imaging Genetics through Meta Analysis-Antisocial Behavior (ENIGMA-ASB) Working Group reported modest acceleration in brain-predicted age among adolescents with CD; however, this effect was specific to adolescent-onset cases (42). Given that elevated CU traits are more strongly associated with childhood-onset CD and more persistent antisocial trajectories (3), these findings raise the possibility that distinct neurodevelopmental pacing may characterize CU-enriched profiles.

Functionally, the SN, anchored in the anterior insula and dorsal anterior cingulate, typically facilitates transitions between internally oriented (DMN) and externally focused (FPN) states and enables adaptive attention to socioemotional cues. Increasing DMN-SN segregation with age is thus expected in typical development, supporting growing efficiency in shifting between self-referential and externally salient processing. However, in youth with elevated CU traits, the pace at which this segregation occurs may lag behind typically developing peers, possibly due to early reductions in DMN intrinsic coherence, which may limit its coordination with the SN and delay typical network maturation and differentiation. By mid-adolescence, these youth seem to show an accelerated segregation that may then reduce the SN’s capacity to re-engage the DMN during socially salient, empathic contexts. This could lead to diminished integration of emotional salience with self-referential processing, paralleling a stabilization of empathic insensitivity and CU traits during adolescence.

Interestingly, this neural pattern may also relate to a socioaffective phenomenon noted in the CU literature, wherein affective empathy (e.g., emotional reactivity) deficits are found to emerge early and persist in those with elevated CU traits, but cognitive empathy (e.g., facial emotion recognition) appears deficient earlier in development, possibly as a function of emotional hyporeactivity, but eventually catches up or even outpaces that of typically developing peers by adolescence (39,43). These improvements are theorized to stem from instrumental social learning, wherein adolescents with elevated CU traits become increasingly motivated to attend to and learn others’ emotions for goal-directed reasons (e.g., social dominance, manipulation), rather than genuine affective concern. Thus, the observed acceleration in DMN-SN segregation may present an underlying mechanism of late developmental compensation: The social brain reorganizes rapidly, achieving mature specialization, but in a way that supports strategic rather than empathic social cognition. Importantly though, future replication of this finding and its theoretical implications is needed to more confidently assess its neurodevelopmental and clinical relevance.

### Prior Work

We found no associations between CU traits and within- or between rsFC of the FPN. This is consistent with the premise that CU traits primarily stem from problems in socioaffective rather than executive control networks, at least in community samples that may reflect lesser contributions or confounding by comorbidities or environmental factors (e.g., impaired cognitive functioning, influences on neuronal maturation by substance misuse and/or trauma exposure) (7,24). Thus, any association with FPN connectivity may reflect a more severe, comorbid profile of CU traits rather than deficits innate to CU traits themselves.

Despite well-documented sex differences in the prevalence of elevated CU traits and in some aspects of neural maturation, we found no sex differences in the association between CU traits and rsFC associations. The large sample size and repeated assessments provide strong evidence that CU-related alterations in DMN and age-related DMN-SN connectivity are broadly shared across sexes. This aligns with emerging meta-analytic work (44) indicating that core socioemotional features of CU traits are similar in males and females and past rsFC work examining associations with CU traits (17,23). These findings suggest a largely common neurodevelopmental pathway, supporting intervention strategies that target socioaffective processes in both sexes.

### Limitations

Several limitations warrant consideration. First, CU traits were measured using a brief parent-report scale. Although this scale is validated and widely used, such measures may underestimate adolescents’ internal emotional experiences and are vulnerable to reporter bias. Multi-informant approaches, including youth self-reports or clinician-rated instruments, would enhance construct validity. Second, the ABCD cohort is community based and not selected for any specific psychopathology; thus, base rates of severe or elevated levels of CU traits were limited, reducing generalizability to clinical populations where neural differences may be more pronounced. Third, the current study focused on rsFC as an index of intrinsic network organization rather than task-evoked responses. While task-based measures would provide convergent evidence linking neural connectivity patterns to real-time behavioral performance, such data were beyond the scope of this analysis. Future work should examine whether rsFC differences predict performance on tasks of empathic and socioemotional processing. Fourth, our hypothesis-driven approach focused on 3 large-scale cortical networks based on established theoretical models of CU traits. While this provides interpretive clarity and reduces multiple comparison burden, it limits detection of connectivity differences in other cortical networks and does not explicitly examine specific subcortical structures known to be relevant to CU neurobiology. Additionally, network-level analyses cannot determine whether observed effects are driven by specific cortical nodes versus distributed network properties. Complementary approaches, including whole-brain exploratory analyses and region-based connectivity investigations integrating cortical-subcortical circuits, could reveal unanticipated patterns and identify specific neural substrates. However, such exploratory work requires careful attention to replication and multiple comparison correction. Such limitations present opportunities for important next steps in this research.

It is also important to note that, though effects are small, the observed associations are consistent with brain-behavior correlations typical of large neuroimaging studies (26), suggesting that, while CU traits appear related to aberrations in network-level connectivity, these effects explain only a proportion of individual variance. More comprehensive models that include functional neural reactivity as well as gene and environment considerations, including low parental warmth (38), may provide a fuller picture of developmental correlates to CU traits.

### Conclusions

The current findings offer a developmentally grounded perspective on the neural correlates of CU traits. Findings suggest that CU traits are linked to alterations in large-scale network maturation across adolescence. Youth with elevated CU traits show persistent within-DMN hypoconnectivity and, by mid-adolescence, an age-related decline in DMN-SN connectivity, reflecting a delayed but accelerated segregation of socioaffective networks. These patterns may underlie the characteristic symptoms of youth with elevated CU traits. Further, this developmentally dynamic finding may parallel behavioral evidence that youth with elevated CU traits display delayed but later enhanced cognitive empathy skills, potentially reflecting compensatory, instrumental sociocognitive adaptations rather than genuine affective engagement. By situating CU-related neural differences within a developmental framework, this study highlights adolescence as a period of heightened plasticity and opportunity for intervention.

## Supplementary Material

Supplemental Note and Tables S1–S2

Key Resources Table

## Footnotes

a To further address this issue, separate supplemental models adjusted for co-occurring aggression problems (31) and the presence of a baseline-assessed lifetime diagnosis of childhood-onset CD with the Kiddie Schedule for Affective Disorders and Schizophrenia (45), in the case that early engagement in antisocial behavior, or contemporaneous aggression problems, better account for variance in rsFC outcomes. Finally, to account for potential global morphometric influences on rsFC, sensitivity analyses included total cortical surface area as an additional covariate. Total cortical surface area was derived from FreeSurfer-processed structural MRI data (34) using the Desikan-Killiany atlas parcellation (46) and reflected the sum of cortical surface area across all cortical parcels.

b Additional analyses tested whether a quadratic age term (CU × age^2^) better captured developmental moderation than the linear CU × age interaction. Including the quadratic term did not significantly improve model fit (likelihood ratio test: χ^2^ = 2.83, *p* = .24), and no interaction between CU traits and quadratic age was significant. Further, although the linear CU × age interaction showed marginal attenuation when the quadratic term was included (*p* = .07), effect sizes and developmental patterning remained consistent (*B* [SE] = −0.0002 [0.0001], β [SE] = −0.0050 [0.0028]). These results indicate that the linear developmental model provides an adequate and parsimonious representation of age-related moderation in this sample.

c The modest attenuation of the association between CU traits and DMN connectivity with ADHD and anxiety included (β = −0.0182) was not further reduced when CD was added (β = −0.0190), despite CD being more strongly correlated with CU traits. This pattern may reflect statistical suppression, in which CD symptoms help isolate CU-specific variance [see Frick (38) and Kemp *et al*. (39)], providing yet more evidence for the need to model CU traits separately from general conduct problems for inclusion as a specifier for CD.

d Results from supplemental sensitivity models adjusting for co-occurring aggression problems, lifetime diagnosis of childhood-onset CD, and cortical surface area are reported in Tables S1 and S2. The pattern of effects and significance of findings (i.e., main effect of CU traits on within-DMN rsFC and CU traits × age interaction on between-network DMN-SN rsFC) remained unchanged across these tests.
