# Supplementary material for: Aberrant Resting-State Functional Connectivity Associated With Callous-Unemotional Traits From Late Childhood Through Late Adolescence
Source: Biol Psychiatry Glob Open Sci. 2026 May 12;6(5):100756. doi: 10.1016/j.bpsgos.2026.100756 (PMC13321020; doi:10.1016/j.bpsgos.2026.100756)
Supplement: Supplemental Note and Tables S1–S2 [file mmc1.pdf]

SUPPLEMENTARY INFORMATION

Aberrant Resting-State Functional Connectivity Associated With Callous-Unemotional Traits From Late Childhood Through Late Adolescence

Kemp *et al.*

Supplemental Note: Validity for the ABCD 4-Item Measure of Callous-Unemotional Traits

Previous work has shown this brief CU composite has acceptable internal consistency, measurement invariance across sex and race/ethnicity, and expected associations with externalizing behavior and reduced prosociality, both in ABCD and an independent replication sample (1). In the current study and across timepoints, CU scores showed moderate-to-strong associations with externalizing (r = .49), rule-breaking (r = .46), and aggression symptoms (r = .46), more modest associations with ADHD symptoms (r = .32), and weaker associations with anxiety symptoms (r = .16), all of which is consistent with prior validation work (2). In addition, the 4-item CU score was correlated (r = .51) with an abbreviated (12-item) version of the Inventory of Callous-Unemotional Traits (ICU) collected in the ABCD-Social Development Substudy (baseline n = 2,426) and showed associations of similar magnitude with relevant validators (e.g., r = .45 for 12-item ICU score and aggression problems).

Supplemental Table S1. Main effects of callous-unemotional (CU) traits on resting state functional connectivity (rsFC) outcomes in the presence of additional sensitivity covariates

Outcome	Covariates	*B* (*SE*)	β (*SE*)	*p*-value	*n*youth	*n*obs
DMN	Standard + cortical surface area	-.0009 (.0003)	-.0210 (.0062)	.001*	10530	22666
FPN		.0003 (.0003)	.0065 (.0063)	.299		
SN		.0005 (.0006)	.0064 (.0066)	.332		
DMN-FPN		.0002 (.0002)	.0059 (.0068)	.387		
DMN-SN		-.0002 (.0003)	-.0044 (.0067)	.509		
FPN-SN		-.0002 (.0003)	-.0043 (.0066)	.514		
DMN	Standard + comorbid ADHD, anxiety, & aggression symptoms	-.0009 (.0003)	-.0218 (.0069)	.002*	10530	22660
FPN		.0002 (.0003)	.0042 (.0070)	.550		
SN		-.0000 (.0006)	-.0005 (.0074)	.942		
DMN-FPN		-.0000 (.0002)	.0018 (.0074)	.807		
DMN-SN		-.0004 (.0003)	-.0099 (.0074)	.182		
FPN-SN		-.0002 (.0003)	-.0050 (.0073)	.495		
DMN	Standard + comorbid ADHD, anxiety + presence of childhood-onset CD	-.0007 (.0003)	-.0167 (.0064)	.009*	10530	22660
FPN		.0005 (.0003)	.0112 (.0065)	.087		
SN		.0006 (.0006)	.0069 (.0069)	.312		
DMN-FPN		.0002 (.0002)	.0056 (.0071)	.427		
DMN-SN		-.0004 (.0003)	-.0090 (.0070)	.199		
FPN-SN		-.0000 (.0003)	-.0011 (.0068)	.871		

Note. DMN = default mode network; FPN = frontoparietal network; SN = salience network; obs = observation; CD = conduct disorder. Unstandardized (*B*) and standardized (β) estimates and

standard errors (*SE*) are presented for the main effect of CU traits. “Standard” covariates = youth age, sex, pubertal status, study timepoint, MRI scanner type, and head motion. Random effects included participant ID nested within family ID nested within assessment site.

* significant at $p < .05$

Supplemental Table S2. Interactions effects of callous-unemotional (CU) traits and either youth age or sex on resting state functional connectivity (rsFC) in the presence of additional sensitivity covariates

**Predictor**	**Outcome**	**Covariates**	***B*** **(*SE*)**	**β (*SE*)**	***p*-value**	***n*youth**	***n*obs**
CU*Age	DMN	Standard + cortical surface area	-.0002 (.0001)	-.0035 (.0023)	.117	10530	22666
CU*Female			-.0000 (.0005)	.0005 (.0124)	.965		
CU*Age	FPN		-.0000 (.0001)	-.0006 (.0023)	.777		
CU*Female			.0002 (.0005)	.0043 (.0127)	.737		
CU*Age	SN		-.0001 (.0002)	-.0006 (.0024)	.800		
CU*Female			.0006 (.0011)	.0068 (.0133)	.607		
CU*Age	DMN-FPN		-.0000 (.0001)	-.0006 (.0025)	.823		
CU*Female			.0000 (.0004)	.0011 (.0137)	.934		
CU*Age	DMN-SN		-.0003 (.0001)	-.0068 (.0025)	.005*		
CU*Female			-.0001 (.0006)	-.0018 (.0135)	.892		
CU*Age	FPN-SN		-.0001 (.0000)	-.0017 (.0024)	.478		
CU*Female			.0006 (.0006)	.0146 (.0132)	.270		
CU*Age	DMN	Standard + comorbid ADHD, anxiety, aggression symptoms	-.0002 (.000)	-.0036 (.0023)	.122	10530	22660
CU*Female			-.0000 (.0005)	-.0008 (.0127)	.947		
CU*Age	FPN		-.0000 (.0000)	-.0006 (.0023)	.787		
CU*Female			.0002 (.0005)	.0045 (.0130)	.727		
CU*Age	SN		-.0000 (.0002)	-.0002 (.0025)	.949		
CU*Female			.0007 (.0011)	.0083 (.0137)	.546		
CU*Age	DMN-FPN		-.0000 (.0000)	-.0003 (.0025)	.897		

CU*Female			.0000 (.0004)	.0029 (.0137)	.834		
CU*Age	DMN-SN		-.0003 (.0001)	-.0065 (.0025)	.009*		
CU*Female			-.0001 (.0006)	-.0031 (.0136)	.818		
CU*Age	FPN-SN		-.0000 (.0001)	-.0018 (.0024)	.456		
CU*Female			.0007 (.0006)	.0158 (.0134)	.241		

CU*Age	DMN	Standard + comorbid ADHD, anxiety + presence of childhood-onset CD	-.0002 (.0001)	-.0039 (.0023)	.088	10530	22660
CU*Female			-.0001 (.0005)	-.0020 (.0124)	.872		
CU*Age	FPN		-.0000 (.0001)	-.0011 (.0023)	.630		
CU*Female			.0002 (.0005)	.0036 (.0127)	.778		
CU*Age	SN		-.0001 (.0002)	-.0007 (.0024)	.789		
CU*Female			.0006 (.0011)	.0070 (.01)	.598		
CU*Age	DMN-FPN		-.0000 (.0001)	-.0005 (.0025)	.833		
CU*Female			.0001 (.0004)	.0029 (.0137)	.835		
CU*Age	DMN-SN		-.0003 (.0001)	-.0065 (.0025)	.009*		
CU*Female			-.0001 (.0006)	-.0027 (.0135)	.844		
CU*Age	FPN-SN		-.0001 (.0001)	-.0020 (.0024)	.398		
CU*Female			.0007 (.0006)	.0152 (.0133)	.252		

Note. DMN = default mode network; FPN = frontoparietal network; SN = salience network; obs = observations; CD = conduct disorder. Unstandardized (*B*) and standardized (β) estimates and standard errors (*SE*) presented for interactions between CU traits and age or sex (Female). "Standard" covariates = youth age, sex, pubertal status, study timepoint, MRI scanner type, and head motion. Random effects = participant ID within family ID within assessment site.

* significant at $p < .05$

Supplemental References

1. Hawes SW, Waller R, Thompson WK, Hyde LW, Byrd AL, Burt SA, et al. Assessing callous-unemotional traits: development of a brief, reliable measure in a large and diverse sample of preadolescent youth. Psychol Med. 2020 Feb;50(3):456–64.

2. Cardinale EM, Marsh AA. The Reliability and Validity of the Inventory of Callous Unemotional Traits: A Meta-Analytic Review. Assessment. 2020 Jan;27(1):57–71.
